# Variables Affecting the Recovery of *Acanthamoeba* Trophozoites

**DOI:** 10.3390/pathogens10020221

**Published:** 2021-02-18

**Authors:** Monica J. Crary, Rhonda Walters, Paul Shannon, Manal M. Gabriel

**Affiliations:** R&D Microbiology, Alcon Research, LLC, Fort Worth, TX 76134, USA; rhonda.walters@alcon.com (R.W.); stephen.shannon@alcon.com (P.S.); manal.gabriel@alcon.com (M.M.G.)

**Keywords:** *Acanthamoeba*, contact lens care, efficacy testing, trophozoites

## Abstract

While the results of *Acanthamoeba* testing have been extensively published, laboratories conducting such testing are left to develop their own methods in the absence of a standardized methodology. The wide disparity of methods has resulted in equally inconsistent reported results for contact lens care (CLC) products. This study’s objective was to determine the source of these discrepancies by evaluating basic *Acanthamoeba* biology and their impact on antimicrobial efficacy testing, including the ability of a recovery method to stimulate a single trophozoite to proliferate. Antimicrobial efficacy testing was conducted using well-published *Acanthamoeba* strains, storage conditions, and growth-based recovery methods. To identify variables that influence results, test solutions with low *Acanthamoeba* disinfection rates were utilized to prevent differences from being masked by high log reductions. In addition, single-cell proliferation assays were executed to understand the growth requirements to stimulate trophozoite propagation in two recovery methods. These studies indicated that both nutrient density (>10^6^ CFU) and the length of plate incubation (at least 14 days) could significantly influence the accurate recovery of trophozoites. Together, this study emphasizes the need to understand how *Acanthamoeba* trophozoites biology can impact test methods to create divergent results.

## 1. Introduction

*Acanthamoeba* keratitis is a blinding eye infection, most often associated with contact lens users and poor contact lens care compliance [1,2,3,4,5]. *Acanthamoeba*, a biphasic protist, is found ubiquitously in nature with a preference for soil–water interfaces [6,7,8]. As a bacterivore, *Acanthamoeba* rarely causes opportunistic infections in humans and animals but has been associated with keratitis outbreaks in the United States and England [9,10,11,12,13,14,15]. *Acanthamoeba* keratitis stories have been widely reported in mainstream news, highlighting high-risk behaviors such as swimming with contact lenses and poor contact lens hygiene [16,17,18,19]. Since 2009, the FDA and other regulatory bodies have been urged to include *Acanthamoeba* as a standard microorganism to evaluate the antimicrobial efficacy of new contact lens care products [20]. Previous *Acanthamoeba* outbreaks have been associated with ineffective anti-*Acanthamoeba* activity of a contact lens care (CLC) product [21], and for this purpose, it is common for CLC manufacturing companies to highlight their *Acanthamoeba* efficacy for marketing. Likewise, other keratitis outbreaks, such as the *Fusarium* outbreak in 2005, were also a result of poor antimicrobial efficacy of CLC products [22]. Current requirements for registering a new CLC product require significant antimicrobial efficacy against common eye pathogens with representative species of bacteria, yeast, and mold [23]. *Acanthamoeba* has not been included in current standards, and no regulatory requirement exists for CLC products to show efficacy against any form of *Acanthamoeba*. Standardization of *Acanthamoeba* testing is challenging due to its unusual methods of cultivation and recovery requirements, which differ from most standard microbiological methods [23]. 

Without a regulatory standard, CLC manufacturing companies are left to develop their own methods for testing their products for antimicrobial efficacy against *Acanthamoeba*. *Acanthamoeba* biology further complicates testing by having two phases, the infective, mobile trophozoite phase and the drug-resistant, inert cyst phase [24]. These two forms possess radically different biocidal resistance and require special cultivation to generate homogenous populations essential for understanding the efficacy of a CLC product [25,26,27]. Finally, numerous variables such as recovery, cultivation, nutrient source, *Acanthamoeba* strain and source, neutralization [27,28,29], and quantification method have led to divergent methods that rarely, if ever, result in similar antimicrobial efficacy results [27,28,30,31,32,33]. As such, any antimicrobial efficacy testing done with *Acanthamoeba* must be critically scrutinized to evaluate the validity of the results. Rapid methods such as Alamar blue and flow cytometry have yet to gain traction as a suitable alterative as results based on percent viability rarely agree with traditional methods of efficacy testing (i.e., log reductions based on serial dilutions and growth recovery [34,35,36,37]), though recently, we have described a propidium iodide staining method that showed a strong correlation with recovery-based testing [38].

The current *Acanthamoeba* antimicrobial efficacy test most prevalent in the literature is loosely based on FDA 510k [39] and International Standards Organization (ISO) 14729 [23], wherein a set concentration of *Acanthamoeba* is inoculated into CLC aliquots and the CLC solutions are allowed to disinfect for the general time a contact lens would be expected to soak in the solution. The CLC is then neutralized based on its active ingredients, and *Acanthamoeba* is recovered from the solution. The surviving *Acanthamoeba* concentration is then determined. Each step of this process has numerous iterations that could ultimately affect the results, and efforts have been ongoing for several years to standardize the testing of *Acanthamoeba* disinfection efficacy of CLC products. The FDA, along with the American Academy of Ophthalmology, American Academy of Optometry, American Optometric Association, the Contact Lens Association of Ophthalmologists, and Contact Lens Institute conducted a Product Workshop to discuss the current gap in *Acanthamoeba* testing in 2009 [40,41]. Since then, no antimicrobial efficacy standards have been adopted, although numerous labs including academic, industrial, and governmental labs have published papers on the subject [27,42,43,44,45,46,47,48]. In 2015, an international standard (ISO 19045) was published to address the potential for CLC products to induce *Acanthamoeba* encystment [49]. This standard recommends all CLC manufacturers to assess their CLC products for their ability to induce *Acanthamoeba* trophozoites to encyst within 24 h [49]. The ability to induce encystment was found to be the major risk factor for the outbreak associated with Advanced Medical Optics Complete MoisturePlus Multi-Purpose Solution where propylene glycol was found to stimulate encystment [12]. 

In this paper, we examine the impact of basic *Acanthamoeba* biology and the potential pitfalls that prevent the development of robust testing methods for evaluating *Acanthamoeba* trophozoites. We also emphasize how *Acanthamoeba* can be set to deliver differential results even when using laboratory strains. Finally, we make general recommendations around *Acanthamoeba* testing and highlight techniques that laboratories can use to ensure their methods are reproducible and valid.

## 2. Results

### 2.1. Antimicrobial Efficacy Testing

#### 2.1.1. Stand-Alone Procedure

**Figure 1:**
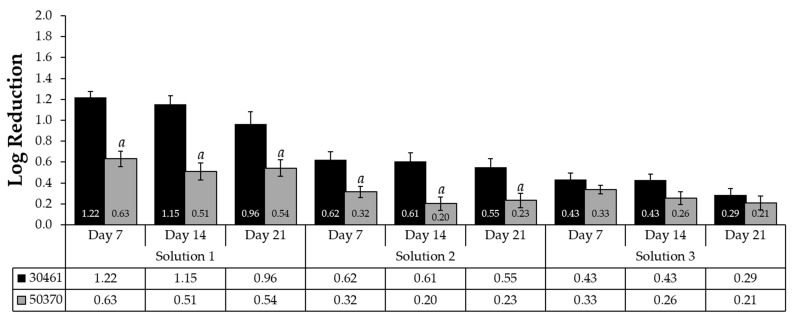
Strain ATCC 30461 demonstrates a significantly greater log reduction than strain ATCC 50370. Mean ± SE comparison of antimicrobial activity for three test solutions per *Acanthamoeba* strain as determined on Day 7, 14, and 21 plate reads. Means reflect combined recovery method and source data. *a: p* < 0.05 vs. 30461 of the same solution and day. *n* = 36/group.

The susceptibility of the two strains of *Acanthamoeba* to common CLC biocides was evaluated by combining all log reduction data (recovery method and source) to establish the total log reductions for each strain following plate incubation times of Day 7, Day 14, and Day 21 (Figure 1). The impact of longer plate incubations (Day 7 vs. Day 14 vs. Day 21) was also assessed to examine the changes in cell concentration and log reduction over time. *Acanthamoeba* ATCC 50370 had statistically lower log reductions after exposure to Solutions 1 and 2 regardless of the length of plate incubation (*p* < 0.05) (Figure 1). For both *Acanthamoeba* ATCC 30461 and *Acanthamoeba* ATCC 50370, Solution 1 showed the highest antimicrobial efficacy (*p* < 0.01 for all days with both 12-well and 96-well recovery methods). Log reduction changes between Day 7, Day 14, and Day 21 were observed for both strains and all three solutions because of changing cell concentrations (additional positive wells) between incubation time points. The differences in biocide resistance for the two strains are evident, suggesting inherent differences in strain susceptibility. 

**Figure 2:**
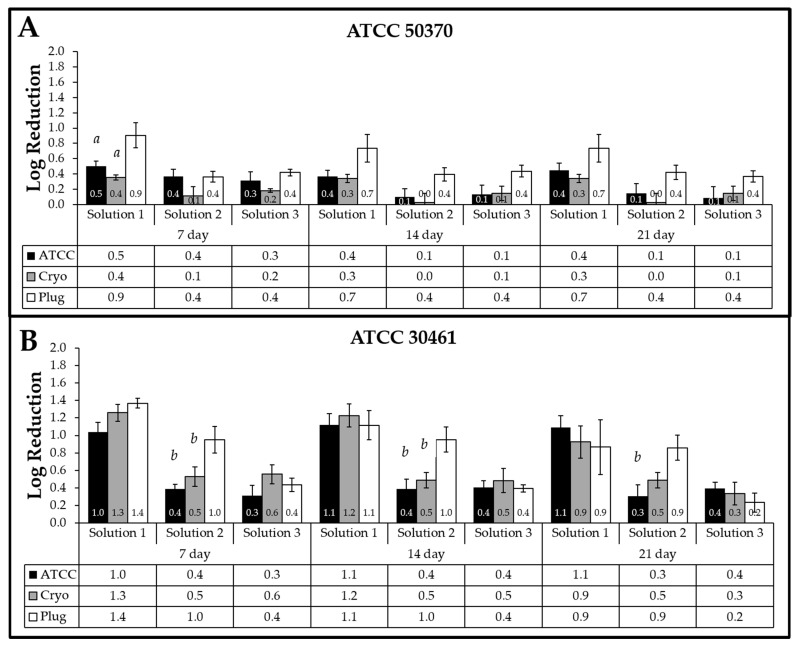
The source (long-term storage method) of *Acanthamoeba* has no impact on biocidal efficacy. Mean ± SE of comparison of log reductions for each test solution against (**A**) *Acanthamoeba* ATCC 50370 and (**B**) *Acanthamoeba* ATCC 30461 trophozoites by long-term maintenance method for Day 7, 14, and 21 plate reads. *a*: *p* < 0.05 vs. Solution 1 plug at the same time point, *b*: *p* < 0.05 vs. Solution 2 plug at the same time point. *n* = 6–18/group.

The impact of the three storage methods (ATCC, Cryo, Plug) of long-term *Acanthamoeba* maintenance (“source”, i.e., prepared directly from ATCC aliquots, revival of a cryogenically frozen sample, or use of prepared *Acanthamoeba* plugs) on antimicrobial efficacy were evaluated by comparing the log reductions for each solution/strain regardless of the recovery method (i.e., recovery using a 12-well or 96-well plate). No difference in recovery across all three solutions was seen between any storage methods (ATCC, Cryo, Plug) of *Acanthamoeba* for ATCC 50370 by Day 21 plate read (Figure 2). For ATCC 30461, the only statistical difference that was maintained through Day 21 plate incubation was for Solution 2, where the source “Plug” had a higher log reduction compared to “ATCC”. Together, this experiment indicated that long-term maintenance/storage methods as described did not result in an increase or decrease in biocidal susceptibility for the majority of the solutions tested.

#### 2.1.2. 12-Well Versus 96-Well Recovery Methods 

**Figure 3:**
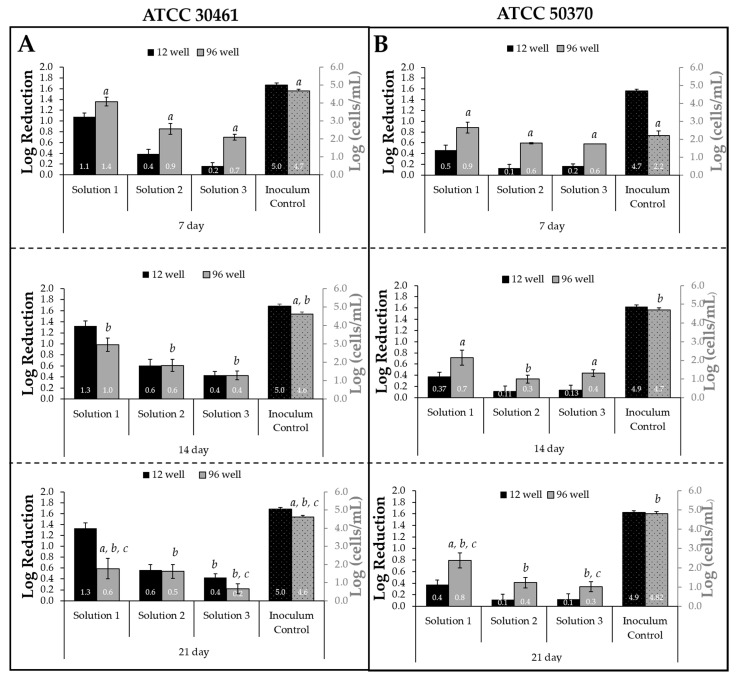
Results show that 96-well recovery changed depending on plate incubation length. Left y-axis: Mean ± SE log reduction of (**A**) *Acanthamoeba* ATCC 30461 and (**B**) *Acanthamoeba* ATCC 50370 trophozoites for the three test solutions per recovery method as determined on Day 7, 14, and 21 plate reads. Right y-axis: total cells/mL (in log) of the paired inoculum control. *a*: *p* < 0.05 vs. 12-well recovery for the same solution at the same day, *b*: *p* < 0.05 vs. Day 7 of the same solution and recovery, *c*: *p* < 0.05 vs. Day 14 of the same solution and recovery. *n* = 12–18/group.

To establish if the two recovery methods (12-well and 96-well) could equally recover unchallenged *Acanthamoeba* trophozoites, the inoculum controls of all tests and sources were compared (Figure 3). For each Test/Source/Strain combination, *Acanthamoeba* trophozoites originated from the same suspension for both inoculum control recovery methods (12-well and 96-well). For both strains, the two methods showed statistically significant differences in the amount of recovery of the Inoculum Controls at Day 7 (Figure 3). For the 96-well, statistically significant differences in recovery were seen for both strains when comparing Day 7 to Day 14 or Day 21. No difference was seen for the 12-well Inoculum Controls when comparing different plate incubation times.

The impact of the two recovery methods on biocide susceptibility was evaluated for both *Acanthamoeba* strains (Figure 3). The log reductions were calculated using the plate concentrations recorded following plate incubations of 7, 14, and 21 days. The 96-well demonstrated different log reductions between Day 7 vs. 14 vs. 21 for both *Acanthamoeba* strains and all three solutions because of changing cell concentrations in either the inoculum controls or the test solution plates (or both). In contrast, no changes in log reduction were observed in the 12-well plate recovery method between Day 14 and Day 21. Statistically significant differences between the two recovery methods were observed for all three solutions for both strains (ATCC 50370 and ATCC 30461) at Day 7. The results obtained on Day 21 were statistically different for the 96-well plate for all Solution/Strain combinations compared to the results obtained on Day 7, whereas the 12-well plate only had a statistically significant difference for ATCC 30461 Solution 3 when comparing Day 7 to Day 21. Together, this indicates that the length of incubation dictated the efficacy obtained in a test.

**Figure 4:**
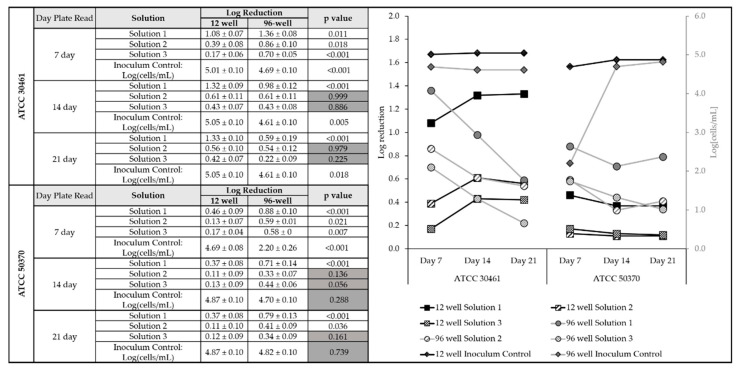
*Acanthamoeba* growth requires at least 14 days to stabilize, with the greatest impact on incubation vs. log reduction results in 96-well method recovery. Left: mean ± SE and associated *p*-values of log reduction by strain, day, and solution. Right: associated visual trend. Left y-axis: Mean log reduction by day of *Acanthamoeba* ATCC 30461 and *Acanthamoeba* ATCC 50370 trophozoites for the three test solutions per recovery method as determined on Day 7, 14, and 21 plate reads. Right y-axis: total cells/mL (in log) of the paired inoculum control. Black lines: 12-well recovery, grey lines: 96-well recovery. *n* = 12–18/group.

The overall trends of how log reductions and inoculum controls changed between increasing plate incubation times were graphed (Figure 4). For ATCC 30461, the 12-well recovery demonstrated an increase in log reductions between Day 7 and Day 14, followed by no change. In contrast, the 96-well recovery decreased between Day 7 and Day 21, dropping from 1.4 to 0.7 for Solution 1. For ATCC 50370, the greatest change was observed in the inoculum control, which increased from 2.2 to 4.8 between Day 7 and Day 21. 

#### 2.1.3. Minimum Plate Incubation Time for Accurate Results

**Figure 5:**
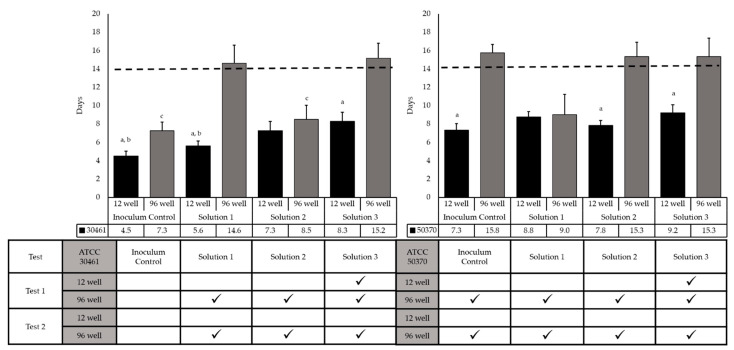
ATCC 30461 recovers faster than ATCC 50370 for both recovery methods, and the 12-well recovery method is the overall fastest recovery method for both strains. Days to final concentration for *Acanthamoeba* ATCC 30461 and ATCC 50370 trophozoites for inoculum controls, and Solutions 1–3, with all sources/test replicates combined. Days to final concentration were determined as the minimum incubation time for the number of positive wells on a recovery method to stop increasing. Dotted line indicates 14 days, which is the standard incubation time for *Acanthamoeba* recovery plates [47,48,50]. *a: p* > 0.05 vs. 96-well of the same strain/solution, *b: p* > 0.0001 vs. 12-well for ATCC 50370 with same solution, *c: p* > 0.0001 vs. 96-well for ATCC 50370 for same solution. Checkmarks in the table indicate where plates/replicates had Day of Final concentration after 14 days, resulting in differences in log reduction between Day 14 results and Day 21 results.

To understand the minimum incubation time needed to obtain accurate final results, the days to final concentration were evaluated for each Solution/Strain/Recovery method combination (Figure 5). For ATCC 30461, the inoculum control recovered statistically faster in the 12-well recovery method than the 96-well recovery method, which was also true for Solution 1 and Solution 3. By comparison, ATCC 50370 recovered statistically faster in the Inoculum Control, Solution 2, and Solution 3 in the 12-well plate versus the 96-well plate. ATCC 30461 had a statistically earlier average day of final concentration for the Inoculum Control for both recovery methods compared to ATCC 50370. The 12-well recovery method reached a final concentration before Day 14 for all strains and solutions tested. 

The table in Figure 5 indicates all Strain/Test Replicate/Recovery Method/Test Solutions, where at least one plate reached a final concentration after Day 14. With ATCC 50370, every test, solution, and inoculum control had 96-well plates reach a final concentration after Day 14.

#### 2.1.4. Single-Cell Proliferation Testing

**Figure 6:**
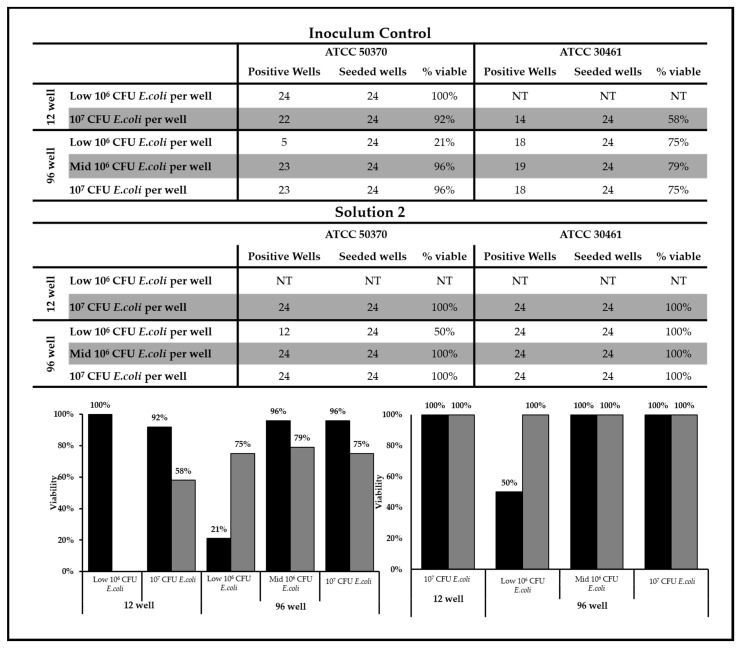
The percent viability of a single trophozoite was dependent on strain and *E.coli* concentration. The number of positive wells following single trophozoite seeding are listed for each strain of *Acanthamoeba* and the different concentrations of *E. coli*. The number of seeded wells indicates the total number of possible positive wells. The percent viability of each strain was graphed to demonstrate the impact of *E. coli* concentration and strain on the recovery of single trophozoites. Black bars: ATCC 50370; grey bars: ATCC 30461. Please see Figure 7 and Figure 8 for statistical analysis of positive wells. NT: Not Tested.

To determine the effect of recovery method and nutrient density (amount of available *Escherichia coli*), single-cell viability was examined in 12-well and 96-well plates using three different *E. coli* densities (low 10^6^, mid-10^6^, and 10^7^ CFU) for up to 65 days. The average viability (percentage of seeded wells demonstrating active trophozoites by day 65) of single trophozoites for both untreated (inoculum control) and Solution 2 samples are shown for both strains of *Acanthamoeba* for all recovery methods and *E. coli* concentrations (Figure 6). *Acanthamoeba* ATCC 50370 showed >90% viability within the inoculum control for both *E. coli* concentrations for the 12-well recovery method, as well as the Mid-10^6^ and 10^7^ CFU *E. coli* per well for the 96-well plate. In contrast, ATCC 30461 showed between 58–79% viability for both recovery methods tested for the inoculum control. Low 10^6^ CFU *E. coli* per well resulted in limited recovery compared to all other nutrient density and recovery methods for ATCC 50370 for both the inoculum control and Solution 2. ATCC 30461 showed lower viability for the inoculum control compared to Solution 2, indicating that the nonviable cells were not available to be selected for seeding in the Single-cell experiment.

**Figure 7:**
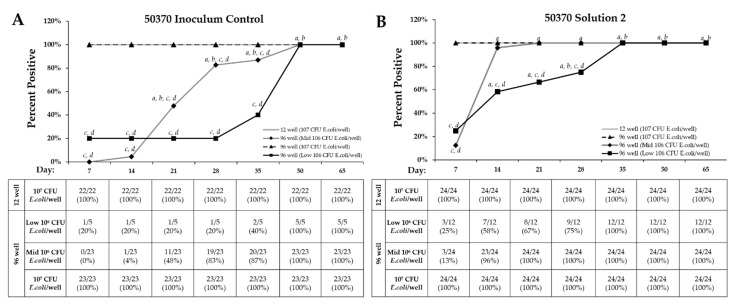
Results show that 96-well recovery and lower *E. coli* density significantly increase the days to final percent positive rates. Day when individual *Acanthamoeba* ATCC 50370 trophozoite wells were identified as positive for each recovery method within (**A**) Inoculum Control and (**B**) Solution 2 treatment. The percentage of positive wells was calculated using the total number of wells positive by Day 65 as the denominator and the positive wells positive on a particular day as the numerator. *a: p* < 0.05 vs. Day 7 within the same recovery and *E. coli* density, *b: p* < 0.05 vs. Day 14 within the same recovery and *E. coli* density, *c: p* < 0.05 vs. 10^7^ CFU/well 96-well, *d: p* < 0.05 vs. 10^7^ CFU/well 12-well.

**Figure 8:**
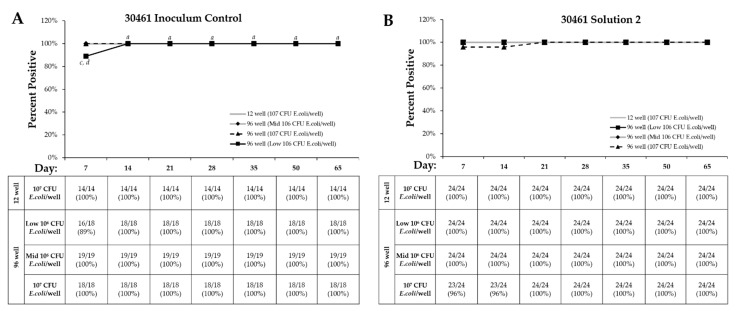
Results show that 96-well recovery and lower *E. coli* density significantly increase the days to final percent positive rates. Days when individual *Acanthamoeba* ATCC 30461 trophozoite wells were identified as positive for each recovery method, within (**A**) Inoculum Control and (**B**) Solution 2 treatment. The percentage of positive wells was calculated using the total number of wells positive by Day 65 as the denominator and the positive wells positive on a particular day as the numerator. *a: p* < 0.05 vs. day 7 within the same recovery and *E. coli* density, *b: p* < 0.05 vs. Day 14 within the same recovery and *E. coli* density, *c: p* < 0.05 vs. 10^7^ CFU/well 96-well, *d: p* < 0.05 vs. 10^7^ CFU/well 12-well.

The percentage of viable wells (percent positivity) was also measured at regular time points between Day 7 and Day 65 to determine the time required to achieve trophozoite activity. For ATCC 50370, a nutrient density of 10^7^ *E. coli* per well, regardless of 12-well or 96-well recovery method, resulted in 100% of positive wells occurring by Day 7 for the inoculum control **(Figure 7**). In contrast, both the Low and Mid-10^6^ *E. coli* per well of the 96-well recovery method continued to have new positive wells through Day 50. For ATCC 30461 inoculum control, all recovery methods demonstrated 100% positivity by Day 14, with the 12-well and the 96-well at Mid-10^6^ and 10^7^ *E. coli* densities producing no additional positive wells after Day 7 (Figure 8). For Solution 2, *Acanthamoeba* ATCC 50370 demonstrated 100% positive wells by Day 7 for the 12-well and the 96-well with 10^7^ CFU *E. coli* (Figure 7). In contrast, the Mid-10^6^ *E. coli* concentration did not reach 100% positivity until Day 21 and Low 10^6^ *E. coli* concentration not until Day 35. ATCC 30461 demonstrated 100% positivity by Day 7 for all recovery methods except 10^7^ CFU *E. coli* (Figure 8). 

#### 2.1.5. Microscopy Observation and Impact of *E. coli* on *Acanthamoeba* Growth 

**Figure 9:**
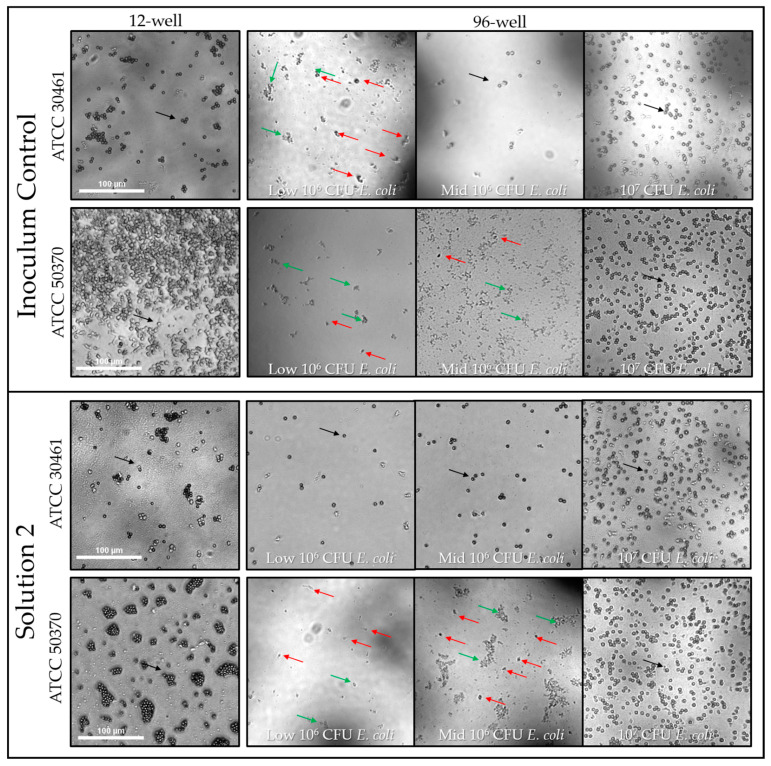
The density of *Acanthamoeba* in positive wells was visualized at the first day a representative well was identified as positive at a designated time point. Inoculum Control wells were evaluated for both strains (ATCC 30461 and ATCC 50370) and captured at 4× magnification. For ATCC 30461, all wells were captured at the Day 7 time point. For 50370, the 12-well and the 10^7^ CFU *E. coli* per well in the 96-well were captured at Day 7, while the two 10^6^ CFU *E. coli* in the 96-well were captured at Day 14. The density of *Acanthamoeba* in positive wells was visualized at the first day a representative well was identified as positive at a designated time point. Solution 2 wells were evaluated for both strains (ATCC 30461 and ATCC 50370) and captured at 4× magnification. All wells were captured at the Day 7 time point. Red arrows indicate only *Acanthamoeba* present, no other *Acanthamoeba* in the field of view; black arrows indicate a representative *Acanthamoeba* in the field. Green arrows indicate *E.coli*. Scale bar = 100 µm, all pictures taken at the same magnification. Reference Figure 6, Figure 7 and Figure 8 for quantification and analysis.

The amount of *E. coli* available for *Acanthamoeba* to consume had a significant impact on trophozoite proliferation. For high-density *E. coli* wells, trophozoite proliferation resulted in *Acanthamoeba* cells numbered in the hundreds to thousands. Proliferation was generally complete by 7 days where all *E. coli* had been consumed and were no longer visible (Figure 9). In many cases, confluent wells contained homogenous populations of cysts after *Acanthamoeba* had completely consumed the available food source (*E. coli*) and then encysted in response to both starvation and trophozoite density. In comparison, in Low-*E. coli* wells, single trophozoites were visible in very low concentrations (<50, often less than 10), and *E. coli* was still visibly present and had not been completely consumed (Figure 9). The impact of *E. coli* density on *Acanthamoeba* trophozoite proliferation was evident for both inoculum controls and Solution 2 (Figure 9).

**Figure 10:**
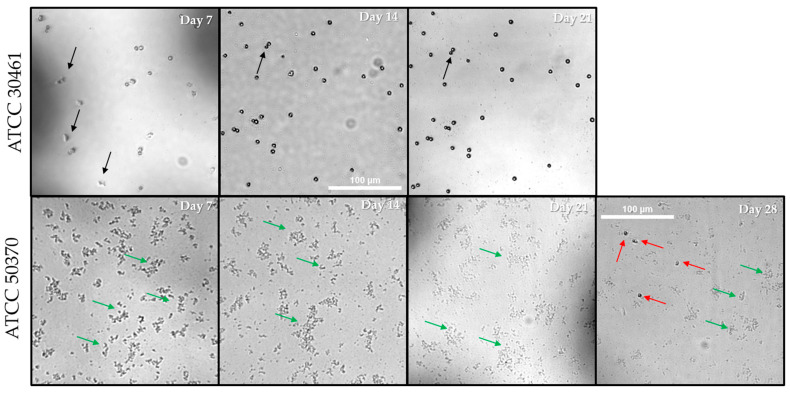
Representative images from ATCC 30461 and ATCC 50370 inoculum controls following seeding with a single *Acanthamoeba* trophozoite on Day 0. Inoculum Control wells from the 96-well containing Mid-10^6^ *E. coli* CFU were evaluated for both strains (ATCC 30461 and ATCC 50370) and captured at 4× magnification. ATCC 50370 was positive on Day 28 as indicated by the red arrows, which show all trophozoites in the field. ATCC 30461 was positive on day 7 with black arrows in Day 7 image indicating trophozoites only. On Day 14 and Day 21, the ATCC 30461 well had fully encysted, and the images show identical cysts at both time points. The single black arrows indicate the same cysts at both time points. Scale bar = 100 µm, all pictures taken at the same magnification. Green arrows indicate *E.coli*. See Figure 6, Figure 7 and Figure 8 for quantification and analysis.

**Figure 11:**
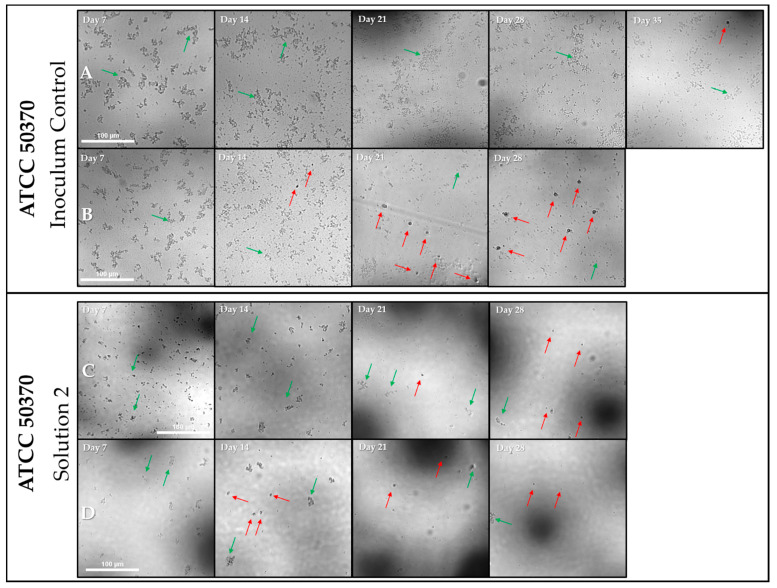
Representative images from ATCC 50370: higher density *E. coli* results in earlier well positivity. (**A**,**B**) Inoculum Control wells from the 96-well containing Mid-10^6^ *E. coli* CFU from ATCC 50370 were captured at 4× magnification. (**A**) was positive at Day 35 as indicated by the arrows, while (**B**) was positive at Day 14. (**C**,**D**) Solution 2 wells from the 96-well containing Low 10^6^ *E. coli* CFU from ATCC 50370 were captured at 4× magnification. (**C**) was positive at Day 21, while (**D**) was positive at Day 14. Red arrows indicate all trophozoites present. Scale bar = 100 µm, all pictures taken at the same magnification. Green arrows indicate *E.coli*. See Figure 6, Figure 7 and Figure 8 for quantification and analysis.

**Figure 12:**
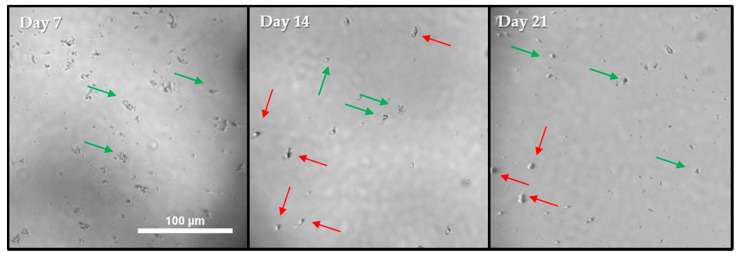
Representative images of a Solution 2 well from a 96-well plate containing Low 10^6^ *E. coli* CFU from ATCC 50370 were captured at 4× magnification. The well was positive at Day 14 as indicated by the red arrows, which indicate all trophozoites present. Scale bar = 100 µm, all pictures taken at the same magnification. Green arrows indicate *E.coli*. See Figure 6, Figure 7 and Figure 8 for quantification and analysis.

The individual wells that failed to show proliferation of trophozoites for long periods were photographed at different intervals. Random wells of each condition were photographed at each time point in order to capture a well becoming positive after prolonged incubation with no previous evidence of proliferation. Within ATCC 30461, only two wells failed to become positive by Day 7 regardless of recovery or *E. coli* density (Figure 8). Thus, a well from the Mid-10^6^ *E. coli* concentration was viewed at Day 7, 14 and 21, despite being positive at Day 7 (Figure 10). For this well, no visible increase in *Acanthamoeba* could be seen, and *Acanthamoeba* were encysted with no visible *E. coli* by Day 7 (Figure 10). Once *Acanthamoeba* were encysted and the *E. coli* were consumed, the well ceased to change, as indicated by the arrows showing identical cysts on Day 14 and Day 21 (Figure 10). In contrast, ATCC 50370 had numerous wells in the 96-well that failed to become positive for extended periods (Figure 8, Figure 10 and Figure 11). Figure 10 and Figure 11 show several example wells of ATCC 50370 failing to proliferate over extended periods. While trophozoites were clearly present as indicated by later positive scoring, the lack of obvious visible trophozoites establishes that *Acanthamoeba* ATCC 50370 can sit dormant and forestall binary fission for many days. Less than 50 trophozoites were visible in wells, but a reduction of *E. coli* is also evident (Figure 12). Similar results were seen for other inoculum control wells with extended periods of no visible proliferation. *Acanthamoeba* trophozoites were not visible for as long as 35 days before low numbers of trophozoites were finally visible with a general decrease in *E. coli* (Figure 11). In wells with extended stasis of *Acanthamoeba*, *Acanthamoeba* failed to proliferate above a density of 20–50 trophozoites (Figure 11). More importantly, even wells that showed proliferation at earlier time points failed to show a significant increase in trophozoite numbers over time (Figure 11). Similar results were observed for ATCC 50370 after exposure to Solution 2, where limited *E. coli* concentration impacted both the speed of proliferation and the quantity of trophozoites (Figure 11). The decrease in *E. coli* was seen following visible proliferation, indicating that significant feeding on the *E. coli* did not occur until visible trophozoites were seen in the well (Figure 11 and Figure 12). Of note, ATCC 50370 struggled to proliferate at both the Low and Mid-10^6^ *E. coli* for both the inoculum control and Solution 2, indicating the phenomena is not isolated to challenged organisms. 

Twenty wells of ATCC 50370 in the 96-well plate failed to show visible proliferation after Day 14, compared to one well of ATCC 30461 (Figure 7 and Figure 8). Of all positive wells seeded in the 96-well plate regardless of concentration (111 wells, Figure 6), 20 wells (representing 18%) failed to proliferate within 14 days (Figure 7 and Figure 8). In contrast, ATCC 30461 had one well of 127 positive wells (or 0.8%) in the 96-well recovery method that failed to proliferate within 14 days (Figure 7 and Figure 8).

## 3. Discussion 

### 3.1. Antimicrobial Efficacy

Many laboratories have developed their own test methods for evaluating efficacy against *Acanthamoeba* trophozoites while waiting for standards to be developed and approved by regulatory bodies. An extensive array of methods have been published in the literature, and just as widespread are the results reported therein [28,29,32,35,42,43,44,45,46,47,48,50,51,52,53,54,55,56,57,58,59]. While standardization of methods is underway by regulatory agencies, our study was meant to ask more fundamental questions around handling, testing, and recovering of *Acanthamoeba* trophozoites than may have been assessed in the past. 

The two strains chosen for this study are well described in the literature [50]. Many laboratories use internal strains [43,58], isolated from the environment or clinical sources [51,59] as well as those available from ATCC [42,44]. This study has shown the importance of standardizing the strains used, as, while both strains were phylogenetically T4 [60], the strains demonstrated different responses to the test formulations and to the test itself. Our recommendation is that utilizing multiple standard *Acanthamoeba* strains with different resistance to biocides is important to fully characterize the efficacy of a product.

The ability of a laboratory to maintain and culture *Acanthamoeba* is critical in producing the homogenous populations of trophozoites needed for use in testing. This study has shown that storage methods do not affect susceptibility to biocides and do not account for the variability seen in the literature. However, long-term passaging in axenic culture has already been established to impact efficacy [52,61,62,63,64]. Genetic drift in long-term cell passages is a known phenomenon [39,65] and is one of the main reasons that most standards require testing within five passages of ATCC [66]. Our recommendation is that laboratories can utilize cryogenic storage or agar plugs to maintain their *Acanthamoeba* stocks (specifically for ATCC 50370 and ATCC 30461) without impacting efficacy.

The efficacy test for *Acanthamoeba* has generally the same steps for most methods that are based on recovery of live organisms: (1) *Acanthamoeba* are inoculated into testing formulations; (2) formulations are sampled and neutralized; (3) samples are serially diluted, and *Acanthamoeba* are recovered on a nutrient source (*E. coli* and *Enterobacter aerogenes*) [67,68,69]; (4) *Acanthamoeba* quantification occurs through a version of calculating a 50% endpoint using Reed and Munch [70], Spearman Karber [71], or Most Probable Number [33]. The recovery format varies from petri dishes [28,51,72] to 96-well plates [27], but the concept is the same, and overall the methods generally mimic the test outlined in ISO 14729 for bacteria, yeast, and mold [23]. Changes in recovery plate format over time reflect the optimization that laboratories are attempting to be able to execute larger tests with fewer resources and consumables.

This study evaluates only two recovery methods, though there are many variations. There are advantages and disadvantages to executing either method. While culturing *Acanthamoeba* is labor-intensive regardless of the method, recovery on 96-well plates requires fewer consumables and is easier to execute with a large number of test samples since a single 96-well plate can hold the equivalent number of samples as six 12-well plates. The agar dispensation into the 12-well requires a more advanced setup compared to 96-well, which can be immediately filled before testing. Execution between the two methods is very different. The 96-well requires both small volume pipetting and the use of multichannel pipetting for sample mixing and serial dilution, which are technical challenges and can easily introduce errors. The 12-well with its large volume pipetting and reliance on vortexing tubes for mixing is less likely to experience unintended errors because of operator error. Additionally, 96-well plates require taping to prevent dehydration, and the handling could easily result in unintended well-to-well transfer of liquid and, by extension, trophozoites. Taping is generally not required for 12-well plates as the agar provides a barrier from over-drying. While incubation is the same for either method, plate reading is easier with the 96-well plate as fewer plates are needed and the 96-well has only one plane of viewing. The 12-well plate can require continuous refocusing to adjust the viewing plane of the microscope due to agar height, which can slow down scoring. This can be mitigated by preventing dehydration of the agar in a humid incubator and precision in the initial filling of the 12-well plates. Despite these seemingly numerous advantages to using a 96-well recovery method, the 96-well plates consistently produced lower densities of *Acanthamoeba* that could easily be misread or time-consuming to identify. The 96-well plate additionally requires significant expertise in recognizing single or low numbers of trophozoites as well as differentiating trophozoites from clumps of *E. coli*. The clumping of *E. coli* can easily mimic trophozoites but is generally only seen in liquid environments, not on agar. Thus, 12-well plate recovery provides a significant advantage by promoting faster, more uniform *Acanthamoeba* growth while also providing an environment that reduces operator variability in identification of *Acanthamoeba* proliferation. 

The conclusion from these investigations is that the major sources of differential results seen in *Acanthamoeba* efficacy testing are incubation time of recovery plates and, by extension, *E.coli* concentration. Our initial study using three different low-efficacy solutions served to evaluate strains, biocide susceptibility, and the impact of storage on efficacy (Figure 1, Figure 2, Figure 3 and Figure 4). This study also assumed the two recovery methods would have similar *Acanthamoeba* recovery results (cell/mL) since both methods are well described in the literature [27,45,50] and have the same basic premise. Our decision to read plates across multiple time points stems from the wide disparity in the literature of when results were recorded [27,29,45,50,73] and wanting to compare speed of recovery for injured and uninjured *Acanthamoeba*. However, we realized while observing plates that there was a vast difference in *Acanthamoeba* response within the plates themselves. For the 12-well plates, significant *Acanthamoeba* proliferation was visible as early as Day 1, with most plates reaching final concentration between Day 3 and Day 7. In comparison, almost no growth or visible trophozoites were seen on Day 1 in the 96-well, regardless of strain, in spite of the significantly smaller surface area we had to view and the expectation that low numbers of trophozoites would be evident sooner in the 96-well compared to the 12-well. The slow attainment of final concentration by the 96-well, even in the inoculum control, was surprising and concerning as was the low density of trophozoites in the 96-well that we observed in the initial Antimicrobial Efficacy study (Figure 5). As our *E. coli* concentration was dictated by the literature, we had not initially considered that to be the main determining factor as to why *Acanthamoeba* proliferated so slowly within the 96-well. Indeed, we assumed that a lower concentration of *E. coli* in the 96-well would be suitable given the working volume (180 μL solutions + 50 μL *E. coli*) [27] compared to the 12-well (1000 μL solution + 100 μL *E. coli*) [47] as well as the available growth surface area (0.32 cm^2^ vs. 3.8 cm^2^, respectively). Nor did we predicate that *Acanthamoeba* would be highly sensitive to the nutrient density such that 2.2 × 10^6^ *E. coli* resulted in such a different *Acanthamoeba* response and confluency compared to 5.0 × 10^6^ *E. coli*. Following the results of our initial experiment, we considered what would drive the observed slower proliferation. The two most likely variables were sample volume, as wells within a 96-well plate may consistently have wells containing only single trophozoites, and nutrient density available for *Acanthamoeba* consumption. The Single-Cell Proliferation assay (Figure 6, Figure 7 and Figure 8) was meant to address both.

### 3.2. Single-Cell Proliferation Assay

As shown by the inoculum control plate images, the relative density of *Acanthamoeba* being defined by the nutrient density was expected: higher *E. coli* density led to greater *Acanthamoeba* proliferation. However, the Single-Cell proliferation assay demonstrated that *Acanthamoeba* could prevent abundant proliferation for extended periods (50 days) based on nutrient density. Equally surprising, the phenomenon was strain-specific as ATCC 50370 consistently demonstrated in the 96-well plate that it found low 10^6^ CFU *E. coli* to be insufficient to trigger confluent proliferation. More critically, small groups of trophozoites could potentially elect to limit proliferation, suggesting a level of communication between trophozoites. In contrast, ATCC 50370 was successful in proliferating in the 12-well plate with low concentrations of *E. coli* (Figure 6 and Figure 7), indicating that the communal proliferation suppression was not communicated in the 12-well plate as it was in the 96-well plate.

**Figure 13:**
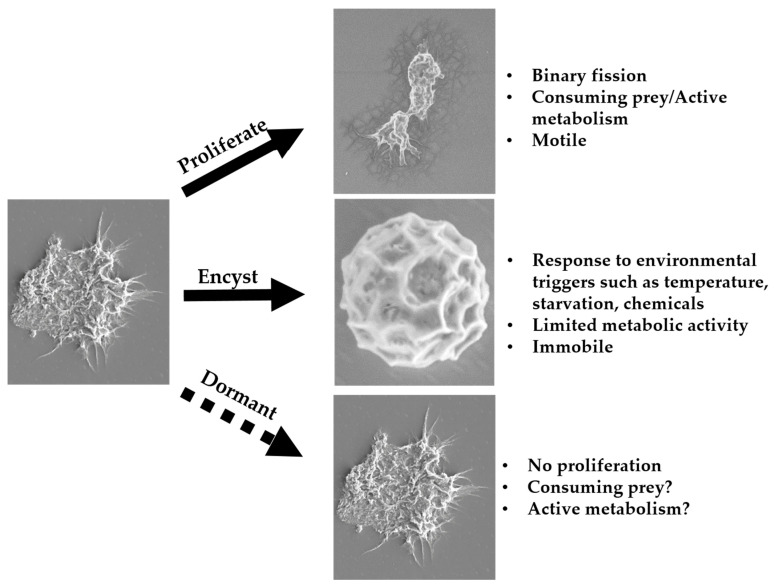
Three potential life cycle paths for *Acanthamoeba* trophozoites, including the new path of dormancy (indicated by the dotted arrow), where *Acanthamoeba* trophozoites display extend proliferation suppression in the presence of a food source.

While *Acanthamoeba* are well-known to respond to chemotactic and intercellular signals [74,75], especially related to encystment [76,77], to our knowledge, this is the first reporting of *Acanthamoeba* communally limiting proliferation in response to nutrient density. From this, we have developed two postulations regarding a trophozoite population in stasis: (1) small numbers (<10) of *Acanthamoeba* trophozoites buried themselves under *E. coli* while feeding, and *E.coli* density provided sufficient cover, preventing detection under the microscope and/or (2) the *Acanthamoeba* limit their proliferation to prevent overgrazing on the limited *E. coli* population until the *E.coli* density reaches a critical point and trophozoite proliferation was triggered to increase the number of cells for eventual encystment. Successive images of the same wells showed how *E. coli* appearance and density changed over time, indicating not only the presence of *Acanthamoeba* but the movement as well. As cysts would be obvious due to autofluorescence [78], the *Acanthamoeba* must have remained trophozoites but were not viewable across many time points and under intense scrutiny as all wells were reviewed carefully at every time point. The trophozoites then become visible once the *E. coli* density decreased to the point that there are no potential visual obstacles provided by the bacteria. We believe that *E.coli* must be at the right concentration to guarantee the proliferation of *Acanthamoeba* trophozoites, which subsequently makes the *Acanthamoeba* so abundant that scoring results are clear to any scientist performing the test. Similarly, we consistently observed <20 trophozoites in 96-well wells that contained low 10^6^ CFU *E. coli* initially, which suggests a communal effort to avoid proliferation (Figure 13). *Acanthamoeba* were also observed to be encysted even when *E. coli* was still present in the low-*E. coli* wells. In contrast, in the high-density *E. coli* wells, *Acanthamoeba* proliferated to confluency and completely consumed the *E. coli*. The intercellular signaling that occurred in the 96-well plates for ATCC 50370, which limited proliferation, did not occur in the 12-well plate as shown by the low *E. coli* results. It is likely that the cell signaling was simply not strong enough to transmit across the larger surface area of the 12-well plate or that the agar itself disrupted the signaling because the water environment is not continuous across the surface throughout incubation. It is a logical conclusion that the different controls and chemotactic responses exist for *Acanthamoeba* based on nutrient density and their own density given their adaptability in the environment [79,80]. Importantly, the fact that a small group of *Acanthamoeba* could contain their population and graze minimally for such extended periods (up to 50 days, as we have demonstrated here) has connotations in all areas of *Acanthamoeba* research, not just efficacy testing. Further, this leads to heightened concern when considering the accuracy of *Acanthamoeba* cyst efficacy testing, where recovery methods have to stimulate both excystment and then proliferation. 

Following the observations in both experiments, we have formulated recommendations regarding the conduction of testing with *Acanthamoeba* trophozoites. While we did not see significant differences between methods at longer plate incubations (Day 21), we recognize that higher efficacy solutions could intensify differences that are seen. If a laboratory desires to use a 96-well recovery format, sufficient concentrations of *E. coli* are needed with the understanding that the threshold of *E. coli* to stimulate proliferation in trophozoites was narrow for the 96-well but not the 12-well plate. Our recommendation is that, when preparing for efficacy testing, laboratories should ensure they are observing confluent proliferation of trophozoites rapidly following recovery. When analyzing conflicting literature results on the efficacy of a solution, we need to examine the recovery method critically, including the nutrient source and density, as well as the day the results were read. Earlier reads prior to 14 days guarantee incorrect results as established by the testing in this study and the literature contains a large number of studies that reported results at Day 7. The impact of incorrect test results may be minimal with low efficacy solutions but with high efficacy solutions, and 1 to 2 wells being incorrectly scored may be the difference between being able to differentiate between formulations. Failure to trigger significant proliferation in trophozoites requires scientists to identify single or low numbers of trophozoites in wells and differentiate them from *E. coli*, such that significant technical expertise is then required to conduct testing. Our recommendation is that laboratories should incubate plates at least 14 days to ensure all wells containing one trophozoite could proliferate to the point of being easily identifiable as positive. 

Lastly, ATCC 30461 was cultured to maximize homogenous populations of trophozoites, including 24-hour scale-ups prior to testing to ensure the health of the cell population. In spite of this, the Single-Cell Proliferation assay demonstrated that a significant nonviable population (up to 40%) was present in ATCC 30461 suspension that was not identifiable under the microscope. This nonviable population was not detected following exposure to Solution 2, suggesting that nonviable cells were lysed in response to biocide exposure. ATCC 50370 did not show a similar nonviable population in the Single-Cell Proliferation experiment and had identical culturing conditions. The impact on antimicrobial efficacy testing in the presence of a nonviable population highlights the need to always base initial *Acanthamoeba* concentrations on recovery plate methods and not on hemocytometer cell counts, which rely on identifying viable *Acanthamoeba* visually. For the Single-Cell Proliferation experiment, all cells selected under the microscope were chosen with criteria meant to identify only healthy cells, including the presence of food cups and pseudopodia, as well as excluding any rounded cells or cells displaying pre-encystment characteristics. Our recommendation is that inoculum control plates should always be used for calculating log reductions rather than relying on the counts from a hemocytometer. The hemocytometer cannot accurately identify viable *Acanthamoeba* and can overestimate the number of *Acanthamoeba* when there is a nonviable amoeba within the initially seeded population, which can lead to artificially inflated antimicrobial efficacy results.

## 4. Materials and Methods 

### 4.1. Antimicrobial Efficacy Testing

#### 4.1.1. Strains and Long-Term Maintenance of *Acanthamoeba* Cultures

Two commonly utilized strains of *Acanthamoeba* (ATCC 50370 and ATCC 30461) from the American Type Culture Collection were evaluated for the antimicrobial efficacy studies [27,45,53]. To evaluate differences in long-term storage methods (“source”), *Acanthamoeba* were maintained/stored over time in three independent ways. First, *Acanthamoeba* were freshly obtained from ATCC prior to testing and immediately put into “master cultures” as described below. This “source” was referred to as “ATCC”. Second, *Acanthamoeba* was obtained from ATCC and maintained in cryogenic storage for approximately 7 years prior to testing and was referred to as “Cryo”. Finally, *Acanthamoeba* was obtained from ATCC and maintained on non-nutrient agar plugs without bacteria as cysts for <1 year, prior to inoculation into “master cultures”. This method of *Acanthamoeba* maintenance was referred to as “Plug”.

#### 4.1.2. Master Cultures and Scale-Up

*Acanthamoeba* from each of the three long-term storage methods were seeded into T75 flasks containing 30 mL of Axenic Culture Media 6 (AC6) to create “master cultures” and were incubated at 28 °C. AC6 is a semidefined axenic medium comprising: Biosate Peptone (BBL; Becton, Dickinson and Company, Oxford, UK) 20.0 g; glucose 5 g; KH2PO4 0.3 g; vitamin B12 10 μg; l-methionine 15 mg per liter of deionized water. The pH was adjusted to 6.5 to 6.6 with 1 M NaOH before autoclaving at 121 °C for 15 min and storage at room temperature for use within 2 months. Flasks were incubated until *Acanthamoeba* were near confluency. Twenty-four hours prior to antimicrobial efficacy testing, master cultures were selectively harvested for adherent trophozoites and seeded into T150 flasks with 75 mL of fresh AC6. Test flasks were incubated at 28 °C overnight. This scale-up ensured homogenous populations of trophozoites.

#### 4.1.3. Trophozoite Harvest and Quantification

On day of test, adherent trophozoites were harvested by pouring off AC6 media and adding 30 mL of ¼ Ringers solution (Oxiod, Basingstoke, UK) to each test flask. Flasks were struck to remove adherent trophozoites and then transferred to 50 mL conical vials. Trophozoites were spun down at 500 g and washed three times with ¼ Ringers solution. Stock trophozoites were adjusted to a concentration of 5.0 × 10^6^ cells/mL for all antimicrobial efficacy testing.

#### 4.1.4. Test Solutions

The three test solutions were prepared with common CLC active ingredients and are listed in Table 1. 

#### 4.1.5. Stand-Alone Procedure

**Figure 14:**
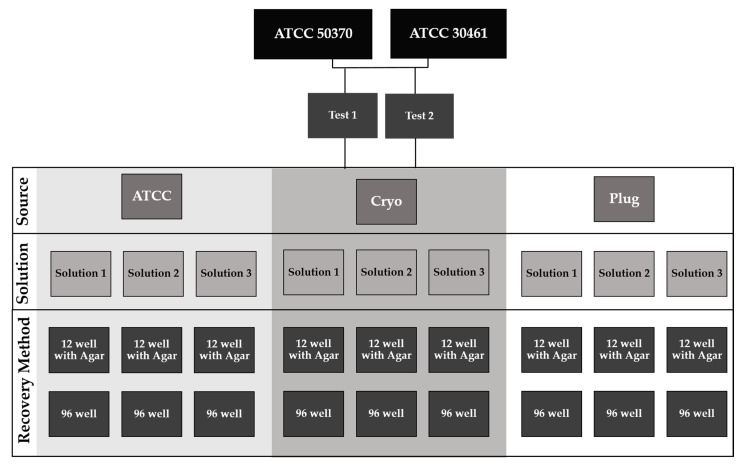
Experimental design system indicating recovery methods and plate counting. Each solution was tested with three independent replicates.

*Acanthamoeba* from each source (ATCC, Cryo, Plug) was inoculated into 10 mL of each solution, in triplicate. Two independent antimicrobial efficacy tests (consisting of equal replicates of all variables) were conducted with separate inoculum controls. Inoculated test solutions were incubated at room temperature for four hours, as a common disinfection time for CLC products. After four hours, *Acanthamoeba* were recovered and plated in two independent methods as described in “12-well versus 96-well Recovery methods and plate scoring” (Figure 14).

#### 4.1.6. 12-Well Versus 96-Well Recovery Methods and Plate Scoring

For each Strain/Source/Solution combination, the test solutions were neutralized and the *Acanthamoeba* was recovered in two independent recovery methods (Figure 14). For the 12-well recovery method, a 12-well plate with 2 mL of non-nutrient agar was prepared in advance and allowed to solidify [47]. Each well was inoculated with 1.0 × 10^7^ CFU/well of *Escherichia coli (E. coli* ATCC 8739*)* before *Acanthamoeba* was added to the plates. One mL of a test solution was put into a tube of 9 mL of lethicin/polysorbate 80 to neutralize the biocides [46,47,54,55,56,57]. Five serial dilutions were created by adding 1 mL of a dilution to 9 mL of ¼ Ringer’s solution. To plate into a 12-well plate, 1 mL of each dilution (−1 through −6) was plated in quadruplicate across four wells. Each replicate/solution/source combination had two 12-well plates to cover dilutions 10^−1^ to 10^−6^ as previously described. All 12-well plates were taped and incubated at 28 °C.

For the 96-well recovery method, 20 µL of the test solution was dispensed into 180 µL of lethicin/polysorbate 80 in quadruplicate across a 96-well plate as described by Kilvington et al. [27]. A multichannel pipette was used to serially dilute the 96-well plates by removing 20 µL of the test solution/lethicin/polysorbate 80 (10^−1^) and dispensing it in 180 µl of ¼ Ringers solution. Successive dilutions were created across the 96-well plate to the 10^−6^. Each well was inoculated with *E. coli* (low 10^6^ CFU/well: 2.2 × 10^6^ CFU) based on methods previously published [27,45,72,81]. Following the addition of *E. coli* to each well, all 96-well plates were taped and incubated at 28 °C.

#### 4.1.7. *Acanthamoeba* Quantification and Days to Final Concentration

At Days 1, 3, 7, 10, 14, and 21, all *Acanthamoeba* recovery plates were scored to determine positive and negative wells. This score (number of positive wells/dilution) determined the surviving *Acanthamoeba* concentration of that plate on that day using Reed Munch 50% endpoint calculations [70]. The day of final concentration was determined based on the day that no additional positive wells were observed in subsequent time points. The day of final concentration was thus identified as the minimum incubation time to achieve final cell concentration results for the test sample or control plate. Using day 14 and day 21 concentrations, log reductions were calculated by subtracting the log survivor cell concentrations for each test solution from the initial log inoculum control concentrations. Student t-tests were conducted on inoculum control concentrations, log reductions, and day of final concentration results using unequal variance.

### 4.2. Single-Cell Proliferation Testing

#### 4.2.1. Culturing Procedure for *Acanthamoeba* Single Cells

Both strains of *Acanthamoeba* (ATCC 50370 and ATCC 30461) were evaluated during single-cell proliferation assays. *Acanthamoeba* strains were cultured, scaled up, and harvested as described for antimicrobial efficacy studies. *Acanthamoeba* were inoculated into either 10 mL of ¼ Ringers solution or Solution 2 (Table 1) at a final concentration of approximately 5.0 × 10^4^ cells/mL. Solution 2 was chosen as it demonstrated intermediate antimicrobial efficacy compared to Solution 1 (highest) and Solution 3 (lowest) (Figure 1). For inoculum control, *Acanthamoeba* was inoculated into ¼ Ringers solution and was plated immediately at Time Zero. For Solution 2, *Acanthamoeba*/Solution 2 samples were incubated for four hours prior to neutralization of the biocide. 

One milliliter of each inoculum control was removed and placed in 9 mL of ¼ Ringers solution and 1 mL inoculated Solution 2 was removed and placed in 9 mL of lethicin/polysorbate 80 to neutralize the biocide. Further dilutions were conducted as needed to adjust the *Acanthamoeba* concentration for single-cell isolation. These steps were intended to mimic antimicrobial efficacy testing such that the *Acanthamoeba* trophozoites were treated identically as they were in the antimicrobial efficacy studies until plating where they were diluted and individual trophozoites placed in separate wells.

#### 4.2.2. Single-Cell Seeding

**Figure 15:**
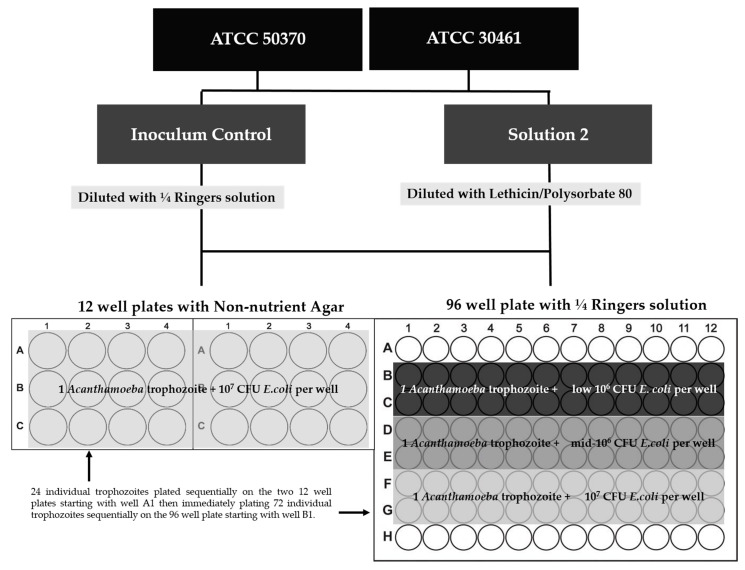
Single-cell experimental design with *E. coli* concentrations.

The inoculated Ringers/*Acanthamoeba* suspensions were poured into petri dishes and viewed under a Nikon Ti microscope at 10× magnification. Individual trophozoites were removed from the suspension using a 10 µL micropipettor. For each strain, single *Acanthamoeba* trophozoites were plated in replicates of 24 for each solution, recovery method, and *E. coli* concentration (Figure 15). Trophozoites were plated sequentially, first 24 replicates into the 12-well with non-nutrient agar, and then 72 replicates into the 96-well plates. All trophozoites for each strain/solution came from the same petri dish to prevent differences in viability between different suspensions. All *Acanthamoeba* were assumed viable during plating.

#### 4.2.3. *E. coli* Different Concentrations Evaluated

*E. coli* was prepared in 100 mL of Tryptic Soy Broth (TSB) at 37 °C shaking for 18–24 h. *E. coli* was harvested by centrifugation and washed with ¼ Ringers solution. Several concentrations of *E. coli* were prepared per published literature depending on the recovery method. For the 96-well, three concentrations were evaluated (Low 10^6^ (2.2 × 10^6^ CFU), Mid 10^6^ (5.0 × 10^6^ CFU), and 1.0 × 10^7^ CFU/well ) based on published methods [27,45], which used a low 10^6^ CFU/mL for the smaller surface area/volume of a 96-well plate. For the 12-well, both strains were fed with 10^7^ CFU/well [47,55,56]. *Acanthamoeba* ATCC 50370 was later evaluated on 12-well plates with low 10^6^ CFU/well due to observations related to ATCC 50370 trophozoite growth in the 96-well plate at low concentrations of *E. coli*. After all single trophozoites had been plated, *E. coli* was added as described in Figure 15, and all plates were taped and incubated at 28 °C (Figure 15).

#### 4.2.4. Microscopy Observation and *Acanthamoeba* Growth Quantification 

Single *Acanthamoeba* trophozoite wells were examined for proliferation on Days 7, 14, 21, 28, 35, 50, and 65. *Acanthamoeba* was incubated beyond 21 days in order to ensure full growth for all strains/recovery methods/*E. coli* concentrations. The experiment was concluded at Day 65 after no change in *Acanthamoeba* growth was observed between Day 50 and Day 65 for any plate. At each time point, all wells were observed and scored as positive or negative. Random wells for all conditions/strains were photographed at each time-point at 4× magnification. As a result, the same wells were imaged across numerous time points to show changes in *Acanthamoeba* growth.

*Acanthamoeba* trophozoites were identified as proliferating, and the well was scored as positive if active motile trophozoites were observed. Wells with single or <10 trophozoites were marked as “low” positive, so the proliferation of an individual well could be observed and evaluated over time. Concentration and appearance of *E. coli* in the wells were also observed as confirmation that active feeding by *Acanthamoeba* was occurring. The number of positive wells for each strain and recovery method condition was determined at each time point. At Day 65, the experiment was concluded and the final total number of positive wells for each recovery method/solution/strain was determined. The percent viability of trophozoites per strain/solution/recovery method/*E. coli* concentration was determined by dividing the total number of positive wells at Day 65 by the original 24 replicate wells. This indicated the general viability of the *Acanthamoeba* going into the study. The percent of positive wells by time point was determined by taking the total wells positive on each day recorded and dividing by the number of positive wells at Day 65. 

### 4.3. Statistical Analysis

Quantifications were calculated as mean ± standard error. Comparisons regarding days to final concentration were analyzed via student t-tests on inoculum control concentrations, log reductions, and day of final concentration results using unequal variance. All other statistical comparisons were calculated by two-way ANOVA or two-way repeated-measure ANOVA followed by a Tukey Test post-hoc analysis. Comparisons were considered significant at a *p*-value of less than 0.05.

## 5. Conclusions 

Our paper is not meant to dictate how all laboratories should conduct testing against *Acanthamoeba* trophozoites; instead, we hope the information in this study assists laboratories in critically evaluating and optimizing their own current methods. As we observed no difference in storage methods, laboratories can utilize a variety of storage methods for their *Acanthamoeba*; however, laboratories should not expect equivalent results between different *Acanthamoeba* strains when testing the same products. A panel of *Acanthamoeba* strains should be utilized when examining efficacy against trophozoites. As *Acanthamoeba* has unpredictable viability, hemocytometer cell counting should not be considered a reliable measure of *Acanthamoeba* trophozoite concentration and recovery-based inoculum controls generated for all testing. Our study supports incubating plates at least through 14 days regardless of recovery format to guarantee rampant proliferation from a single *Acanthamoeba* trophozoite. Finally, laboratories should provide high-density bacteria during recovery to encourage maximum *Acanthamoeba* proliferation and allow for easy, fast identification of *Acanthamoeba* by microscopy.

## Figures and Tables

**Table 1:** Laboratory-prepared Test Solutions with Active ingredients concentrations.

Test Solution	Biocides
Solution 1	Polyquaternium (0.0001%), Polyaminopropylbiguanide (0.0001%)
Solution 2	Polyhexanide (0.0001%)
Solution 3	Polyquaternium-1 (0.001%)

## Data Availability

The data presented in this study are available upon request from the corresponding author. The data are not publicly available due to commercial interests.

## References

[1] Joslin C.E., Tu E.Y., Shoff M.E., Booton G.C., Fuerst P.A., McMahon T.T., Anderson R.J., Dworkin M.S., Sugar J., Davis F.G. (2007). The association of contact lens solution use and *Acanthamoeba* keratitis. Am. J. Ophthalmol..

[2] Marciano-Cabral F., Cabral G. (2003). *Acanthamoeba spp*. As agents of disease in humans. Clin. Microbiol. Rev..

[3] Khan N.A. (2006). *Acanthamoeba*: Biology and increasing importance in human health. FEMS Microbiol. Rev..

[4] Lorenzo-Morales J., Khan N.A., Walochnik J. (2015). An update on *Acanthamoeba* keratitis: Diagnosis, pathogenesis and treatment. Parasite.

[5] Ahearn D.G., Gabriel M.M. (1997). Contact Lenses, Disinfectants, and Acanthamoeba Keratitis.

[6] Preston T.R., Richards H., Wotton R.S. (2001). Locomotion and feeding of *Acanthamoeba* at the water-air interface of ponds. FEMS Microbiol. Lett..

[7] De Jonckheere J.F. (1991). Ecology of *acanthamoeba*. Rev. Infect. Dis..

[8] Kilvington S., White D.G. (1994). *Acanthamoeba*: Biology, ecology and human disease. Rev. Med. Microbiol..

[9] Carnt N., Hoffman J.J., Verma S., Hau S., Radford C.F., Minassian D.C., Dart J.K.G. (2018). *Acanthamoeba* keratitis: Confirmation of the UK outbreak and a prospective case-control study identifying contributing risk factors. Br. J. Ophthalmol..

[10] Joslin C.E., Tu E.Y., McMahon T.T., Passaro D.J., Stayner L.T., Sugar J. (2006). Epidemiological characteristics of a Chicago-area *Acanthamoeba* keratitis outbreak. Am. J. Ophthalmol..

[11] Joslin C.E., Tu E.Y., Shoff M.E., Anderson R.J., Davis F.G. (2010). Shifting distribution of Chicago-area *Acanthamoeba* keratitis cases. Arch. Ophthalmol..

[12] Verani J.R., Lorick S.A., Yoder J.S., Beach M.J., Braden C.R., Roberts J.M., Conover C.S., Chen S., McConnell K.A., Chang D.C. (2009). National outbreak of *Acanthamoeba* keratitis associated with use of a contact lens solution, United States. Emerg. Infect. Dis..

[13] Visvesvara G.S., Shoff M.E., Sriram R., Booton G.C., Crary M., Fuerst P.A., Hanley C.S., Garner M.M. (2010). Isolation, morphologic, serologic and molecular identification of *Acanthamoeba* t4 genotype from the liver of a temminck’s tragopan (tragopan temminckii). Vet. Parasitol..

[14] Valladares M., Reyes-Batlle M., Mora-Peces I., Martín-Navarro C.M., López-Arencibia A., Dorta-Gorrín A., Comyn-Afonso E., Martínez-Carretero E., Maciver S.K., Piñero J.E. (2014). A multisystemic *Acanthamoeba* infection in a dog in tenerife, canary islands, spain. Vet. Parasitol..

[15] Karakavuk M., Aykur M., Şahar E.A., Karakuş M., Aldemir D., Döndüren Ö., Özdemir H.G., Can H., Gürüz A.Y., Dağcı H. (2017). First time identification of *Acanthamoeba* genotypes in the cornea samples of wild birds; is acanthamoeba keratitis making the predatory birds a target?. Exp. Parasitol..

[16] Dorking M.C. (2020). Woman left bed-bound in agonising pain after parasite burrowed into her eye. Yahoo! Sports.

[17] Benyon L. (2020). Touching my contact lens with a wet hand left me blind in one eye. Daily Mail.

[18] Avramova N. (2018). Ongoing outbreak of rare eye infection found among contact lens wearers. CNN.

[19] Lex18 (2018). Woman nearly loses eye after swimming in contacts. Lex18.

[20] Brocious J., Tarver M.E., Hampton D., Eydelman M. (2018). *Acanthamoeba*: An overview of the challenges to the development of a consensus methodology of disinfection efficacy testing for contact lens care products. Eye Contact Lens.

[21] Yoder J.S., Verani J., Heidman N., Hoppe-Bauer J., Alfonso E.C., Miller D., Jones D.B., Bruckner D., Langston R., Jeng B.H. (2012). *Acanthamoeba* keratitis: The persistence of cases following a multistate outbreak. Ophthalmic Epidemiol..

[22] Epstein A.B. (2007). In the aftermath of the fusarium keratitis outbreak: What have we learned?. Clin. Ophthalmol..

[23] EN ISO 14729:2001/A1:2010 (2010). Ophthalmic Optics–Contact Lens Care Products–Microbiological Requirements and Test Methods for Products and Regimens for Hygienic Management of Contact Lenses.

[24] Siddiqui R., Khan N.A. (2012). Biology and pathogenesis of *Acanthamoeba*. Parasit Vectors.

[25] Coulon C., Collignon A., McDonnell G., Thomas V. (2010). Resistance of *Acanthamoeba* cysts to disinfection treatments used in health care settings. J. Clin. Microbiol..

[26] Turner N.A., Russell A.D., Furr J.R., Lloyd D. (2000). Emergence of resistance to biocides during differentiation of *Acanthamoeba castellanii*. J. Antimicrob. Chemother..

[27] Kilvington S., Lam A. (2013). Development of standardized methods for assessing biocidal efficacy of contact lens care solutions against *Acanthamoeba* trophozoites and cysts. Investig. Ophthalmol. Vis. Sci..

[28] Shoff M.E., Eydelman M.B. (2012). Strategies to optimize conditions for testing multipurpose contact lens solution efficacy against *Acanthamoeba*. Eye Contact Lens.

[29] Shoff M., Rogerson A., Schatz S., Seal D. (2007). Variable responses of *Acanthamoeba* strains to three multipurpose lens cleaning solutions. Optom. Vis. Sci..

[30] Kolar S.S.N., Manarang J.C., Burns A.R., Miller W.L., McDermott A.M., Bergmanson J.P.G. (2015). Contact lens care solution killing efficacy against *Acanthamoeba castellanii* by in vitro testing and live-imaging. Contact Lens Anterior Eye.

[31] Nakaminami H., Enomoto K., Yoshimura Y., Onuki T., Nihonyanagi S., Hamada Y., Noguchi N. (2017). Evaluation of in vitro antiamoebic activity of antimicrobial agents against clinical *Acanthamoeba* isolates. J. Ocul. Pharmacol. Ther..

[32] Kobayashi T., Gibbon L., Mito T., Shiraishi A., Uno T., Ohashi Y. (2011). Efficacy of commercial soft contact lens disinfectant solutions against *Acanthamoeba*. Jpn. J. Ophthalmol..

[33] Beattie T.K., Seal D.V., Tomlinson A., McFadyen A.K., Grimason A.M. (2003). Determination of amoebicidal activities of multipurpose contact lens solutions by using a most probable number enumeration technique. J. Clin. Microbiol..

[34] McBride J., Ingram P.R., Henriquez F.L., Roberts C.W. (2005). Development of colorimetric microtiter plate assay for assessment of antimicrobials against *Acanthamoeba*. J. Clin. Microbiol..

[35] Fears A.C., Metzinger R.C., Killeen S.Z., Reimers R.S., Roy C.J. (2018). Comparative in vitro effectiveness of a novel contact lens multipurpose solution on *Acanthamoeba castellanii*. J. Ophthalmic Inflamm. Infect..

[36] Borazjani R.N., May L.L., Noble J.A., Avery S.V., Ahearn D.G. (2000). Flow cytometry for determination of the efficacy of contact lens disinfecting solutions against *Acanthamoeba* spp.. Appl. Environ. Microbiol..

[37] Hughes R., Heaselgrave W., Kilvington S. (2003). *Acanthamoeba* polyphaga strain age and method of cyst production influence the observed efficacy of therapeutic agents and contact lens disinfectants. Antimicrob. Agents Chemother..

[38] Campolo A., Shannon S.P., Crary M.J. (2021). Evaluating alternate methods of determining the antimicrobial efficacy of contact lens care products against *Acanthamoeba* trophozoites. Pathogens.

[39] Seo S.A., Yong T.S., Im K.I. (1992). The maintenance of free-living amoebae by cryopreservation. Korean J. Parasitol..

[40] Clayton-Jeter H.D., FDA (2010). Looking good: Safe use and care of contact lenses. FDA News for Health Care Professioals.

[41] Eydelman M.B., Tarver M.E., Kiang T., Alexander K.Y., Hutter J.C. (2012). The food and drug administration’s role in establishing and maintaining safeguards for contact lenses and contact lens care products. Eye Contact Lens.

[42] Borazjani R.N., Kilvington S. (2005). Efficacy of multipurpose solutions against *Acanthamoeba* species. Contact Lens Anterior Eye.

[43] Connor C.G., Blocker Y., Pitts D.G. (1989). *Acanthamoeba culbertsoni* and contact lens disinfection systems. Optom. Vis. Sci..

[44] Silvany R.E., Dougherty J.M., McCulley J.P., Wood T.S., Bowman R.W., Moore M.B. (1990). The effect of currently available contact lens disinfection systems on *Acanthamoeba castellanii* and *Acanthamoeba polyphaga*. Ophthalmology.

[45] Lonnen J., Heaselgrave W., Nomachi M., Mori O., Santodomingo-Rubido J. (2010). Disinfection efficacy and encystment rate of soft contact lens multipurpose solutions against *Acanthamoeba*. Eye Contact Lens.

[46] Crary M. (2018). Differential Antimicrobial Efficacy of Multipurpose Solutions Against Acanthamoeba Spp Trophozoites.

[47] Crary M.J., Walters R., Bartell J., Gabriel M.M., Kadurugamua J., Catalone B. (2013). Establishing a standard method for evaluating efficacy against *Acanthamoeba*. Investig. Ophthalmol. Vis. Sci..

[48] Fedorko D.P., Brocious J.M., Adams K.D., Hitchins V.M., Hampton D.L., Eydelman M.B. (2018). Optimized protocol for testing multipurpose contact lens solution efficacy against *Acanthamoeba*. Eye Contact Lens.

[49] ISO (2015). Ophthalmic Optics—Contact Lens Care Products—Method for Evaluating Acanthamoeba Encystment by Contact Lens Care Products.

[50] Buck S.L., Rosenthal R.A., Schlech B.A. (2000). Methods used to evaluate the effectiveness of contact lens care solutions and other compounds against *Acanthamoeba*: A review of the literature. CLAO J..

[51] Shoff M.E., Joslin C.E., Tu E.Y., Kubatko L., Fuerst P.A. (2008). Efficacy of contact lens systems against recent clinical and tap water *Acanthamoeba* isolates. Cornea.

[52] Mirjalali H., Niyyati M., Abedkhojasteh H., Babaei Z., Sharifdini M., Rezaeian M. (2013). Pathogenic assays of *Acanthamoeba* belonging to the t4 genotype. Iran. J. Parasitol..

[53] Imayasu M., Tchedre K.T., Cavanagh H.D. (2013). Effects of multipurpose solutions on the viability and encystment of *Acanthamoeba* determined by flow cytometry. Eye Contact Lens.

[54] Gabriel M.M., McAnally C., Bartell J., Walters R., Clark L., Crary M., Shannon S. (2019). Biocidal efficacy of a hydrogen peroxide lens care solution incorporating a novel wetting agent. Eye Contact Lens.

[55] Gabriel M.M., Walters R., McAnally C., Bartell J., Crary M., Catalone B. (2018). Biocidal efficacy of a hydrogen peroxide solution incorporating a novel wetting agent. Contact Lens Anterior Eye.

[56] Crary M.J., Walters R., McAnally C., Gabriel M.M., Shannon S.P. (2018). Differential antimicrobial efficacy of multipurpose solutions against *Acanthamoeba* spp trophozoites. Optom. Vis. Sci..

[57] Gabriel M.M., McAnally C., Walters R., Clark L., Crary M.J., Bartell J., Catalone B. (2015). Biocidal efficacy of a new hydrogen peroxide disinfecting solution against clinical bacterial and yeast isolates, and *Acanthamoeba* species. Investig. Ophthalmol. Vis. Sci..

[58] Niszl I.A., Markus M.B. (1998). Anti-acanthamoeba activity of contact lens solutions. Br. J. Ophthalmol..

[59] del Buey M.A., Cristóbal J.A., Casas P., Goñi P., Clavel A., Mínguez E., Lanchares E., García A., Calvo B. (2012). Evaluation of in vitro efficacy of combined riboflavin and ultraviolet a for *Acanthamoeba* isolates. Am. J. Ophthalmol..

[60] Fuerst P.A., Booton G.C., Crary M.J. (2015). Phylogenetic analysis and the evolution of the 18s rrna gene typing system of *Acanthamoeba*. J. Eukaryot. Microbiol..

[61] Niyyati M., Abedkhojasteh H., Salehi M., Farnia S., Rezaeian M. (2013). Axenic cultivation and pathogenic assays of *Acanthamoeba* strains using physical parameters. Iran. J. Parasitol..

[62] Coulon C., Dechamps N., Meylheuc T., Collignon A., McDonnell G., Thomas V. (2012). The effect of in vitro growth conditions on the resistance of acanthamoeba cysts. J. Eukaryot. Microbiol..

[63] Köhsler M., Leitsch D., Fürnkranz U., Duchêne M., Aspöck H., Walochnik J. (2008). *Acanthamoeba* strains lose their abilities to encyst synchronously upon prolonged axenic culture. Parasitol. Res..

[64] Ferrante A., Bates E.J. (1988). Elastase in the pathogenic free-living amoebae naegleria and acanthamoeba spp.. Infect. Immun..

[65] Mazur T., Hadaś E. (1994). The effect of the passages ofacanthamoeba strains through mice tissue on their virulence and its biochemical markers. Parasitol. Res..

[66] ATCC (2003). Reference strains: How many passages are too many?. ATCC Connect..

[67] Weekers P.H.H., Bodelier P.L.E., Wijen J.P.H., Vogels G.D. (1993). Effects of grazing by the free-living soil amoebae *Acanthamoeba castellanii*, *Acanthamoeba polyphaga*, and *Hartmannella vermiformis* on various bacteria. Appl. Environ. Microbiol.

[68] Wang X., Ahearn D.G. (1997). Effect of bacteria on survival and growth of *Acanthamoeba castellanii*. Curr. Microbiol..

[69] Yousuf F.A., Siddiqui R., Khan N.A. (2013). *Acanthamoeba castellanii* of the t4 genotype is a potential environmental host for *Enterobacter aerogenes* and *Aeromonas hydrophila*. Parasit Vectors.

[70] Reed L.J., Muench H. (1938). A simple method of estimating fifty per cent endpoints. Am. J. Epidemiol..

[71] Hamilton M.A., Russo R.C., Thurston R.V. (1977). Trimmed spearman-karber method for estimating median lethal concentrations in toxicity bioassays. Environ. Sci. Technol..

[72] Kilvington S., Anger C. (2001). A comparison of cyst age and assay method of the efficacy of contact lens disinfectants against *Acanthamoeba*. Br. J. Ophthalmol..

[73] Martin-Navarro C.M., Lorenzo-Morales J., Lopez-Arencibia A., Valladares B., Pinero J.E. (2010). *Acanthamoeba* spp.: Efficacy of bioclen fr one step, a povidone-iodine based system for the disinfection of contact lenses. Exp. Parasitol..

[74] Schuster F.L., Rahman M., Griffith S. (1993). Chemotactic responses of *Acanthamoeba castellanii* to bacteria, bacterial components, and chemotactic peptides. Trans. Am. Microsc. Soc..

[75] Schuster F.L., Levandowsky M. (1996). Chemosensory responses of *Acanthamoeba castellanii*: Visual analysis of random movement and responses to chemical signals. J. Eukaryot. Microbiol..

[76] Du Q., Schilde C., Birgersson E., Chen Z.-h., McElroy S., Schaap P. (2014). The cyclic amp phosphodiesterase rega critically regulates encystation in social and pathogenic amoebas. Cell. Signal..

[77] Moon E.-K., Chung D.-I., Hong Y., Kong H.-H. (2011). Expression levels of encystation mediating factors in fresh strain of *Acanthamoeba castellanii* cyst ests. Exp. Parasitol..

[78] Wang Y.E., Tepelus T.C., Gui W., Irvine J.A., Lee O.L., Hsu H.Y. (2019). Reduction of *Acanthamoeba* cyst density associated with treatment detected by in vivo confocal microscopy in acanthamoeba keratitis. Cornea.

[79] Shabardina V., Kischka T., Kmita H., Suzuki Y., Makałowski W. (2018). Environmental adaptation of *Acanthamoeba castellanii* and *Entamoeba histolytica* at genome level as seen by comparative genomic analysis. Int. J. Biol. Sci..

[80] Guimaraes A.J., Gomes K.X., Cortines J.R., Peralta J.M., Peralta R.H.S. (2016). *Acanthamoeba* spp. As a universal host for pathogenic microorganisms: One bridge from environment to host virulence. Microbiol. Res..

[81] Hughes R., Kilvington S. (2001). Comparison of hydrogen peroxide contact lens disinfection systems and solutions against *Acanthamoeba polyphaga*. Antimicrob. Agents Chemother..

